# Point-Of-Care Capillary Refill Technology Improves Accuracy of Peripheral Perfusion Assessment

**DOI:** 10.3389/fmed.2021.694241

**Published:** 2021-07-22

**Authors:** David C. Sheridan, Robert L. Cloutier, Ravi Samatham, Matthew L. Hansen

**Affiliations:** ^1^Department of Emergency Medicine, Oregon Health & Science University, Portland, OR, United States; ^2^Promedix Inc., Portland, OR, United States; ^3^Department of Dermatology, Oregon Health & Science University, Portland, OR, United States

**Keywords:** perfusion, sepsis, point of care, device, innovation

## Abstract

**Background:** Peripheral perfusion assessment is used routinely at the bedside by measuring the capillary refill time (CRT). Recent clinical trials have shown evidence to its ability to recognize conditions with decreased end organ perfusion as well as guiding therapeutic interventions in sepsis. However, the current standard of physician assessment at the bedside has shown large variability. New technology can improve the precision and repeatability of CRT affecting translation of previous high impact research.

**Methods:** This was a prospective, observational study in the intensive care unit and emergency department at a quaternary care hospital using a non-invasive finger sensor for CRT. The device CRT was compared to the gold standard of trained research personnel assessment of CRT as well as to providers clinically caring for the patient.

**Results:** Pearson correlations coefficients were performed across 89 pairs of measurements. The Pearson correlation for the device CRT compared to research personnel CRT was 0.693. The Pearson correlation for the provider CRT compared to research personnel CRT was 0.359.

**Conclusions:** New point-of-care technology shows great promise in the ability to improve peripheral perfusion assessment performed at the bedside in the emergency department triage and during active resuscitation. This standardized approach allows for better translation of prior research that is limited by the subjectivity of manual visual assessment of CRT.

## Introduction

Approximately 1 million patients present annually to United States emergency departments with sepsis (1). The diagnosis, unfortunately, remains challenging with an 8% increase in mortality for every hour of delayed recognition (2). The mortal consequences of delayed diagnosis creates an imperative for improved early identification and intervention to meet the Surviving Sepsis goals of therapy. One measure that has been used in clinical practice for decades as a marker of perfusion is capillary refill time (CRT). In practice, however, there is a great deal of subjectivity and variability in how providers apply and interpret results of this simple, non-specific marker of oxygen delivery (3). Technology can standardize this measurement making it objective, reproducible and precise. Through a problem-based innovation approach, a point-of-care bedside technology for CRT assessment (Promedix Inc.) has been developed (4); this opens the door to research evaluating CRT's ability to detect sepsis earlier and, hopefully, facilitate earlier diagnosis and more effective resuscitation of septic patients. The objective of this study was to evaluate the performance of a novel medical device to objectively measure CRT compared to both a rigorous manual method and unstructured usual practice as one of the first steps in validating the ability of the device.

## Methods

This was a prospective, observational study performed in a quaternary care university hospital across both an adult intensive care unit (ICU) and the emergency department (ED). The primary outcome of this convenience sample was evaluating the correlation between research personnel measurement of CRT relative to either the point of care technology prototype or provider CRT. This was a parallel study performed as part of a larger study evaluating the utility of CRT in sepsis diagnosis and monitoring of therapeutic interventions. This study had institutional review board approval. Patients and the public were not involved in the design, conduct, or reporting of this study. Patients were eligible if <17 years of age and with a known/suspected infection on intravenous antibiotics for sepsis in the ICU or if there was suspicion for sepsis in the ED. Patients were excluded if they had bilateral hand trauma, were either positive or had a pending COVID-19 test, cirrhosis, inability to consent, diabetic ketoacidosis, chronic liver disease or transplant, chronic kidney disease or transplant, pregnant, or a prisoner.

Following consent, a research assistant assessed CRT by applying manual pressure to a finger on the hand for 3 s and then releasing the pressure. Capillary refill duration was calculated with a chronometer and the stop point determined when the examiner determined the color of the nailbed was back to baseline. The research assistant underwent training by the study team and has extensive clinical research experience, but is not a trained medical practitioner. In the ED group of patients, the research personnel performed CRT manually with a chronometer and then asked the medical provider caring for the patient to assess CRT per standard of care without a chronometer. The current standard of care is to apply pressure to the nailbed to blanch the color and the provider simply counts to themselves until they visually determine the nailbed color is back to normal.

**Figure 1:**
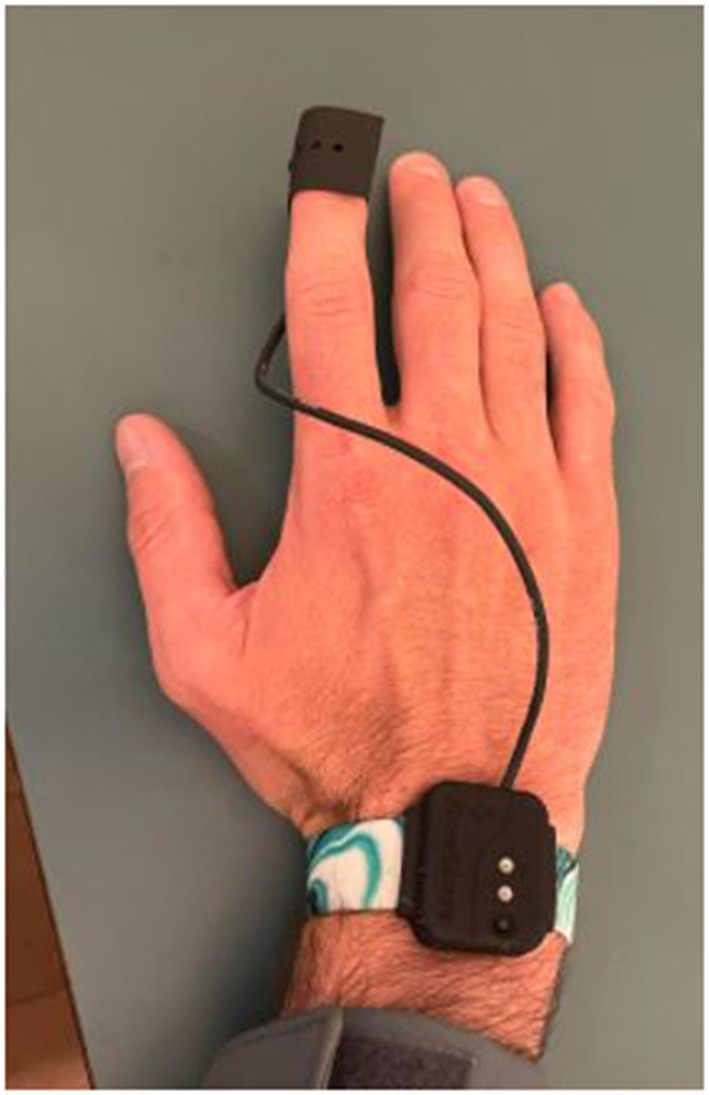
Capillary refill device.

The research personnel performed the same procedure with the point of care technology as manual detection (Promedix Inc.) to measure CRT (4) (Figure 1). The technology is a grooved finger sensor that is applied to the distal phalanx of the patient. It connects *via* Bluetooth technology to an application that contains algorithms that detect manual application of pressure and then calculate exponential decay of the photodiode signal from the light source. The change in capillary blow flow timed with the pressure sensor is able to calculate the duration of capillary refill.

## Results

**Figure 2:**
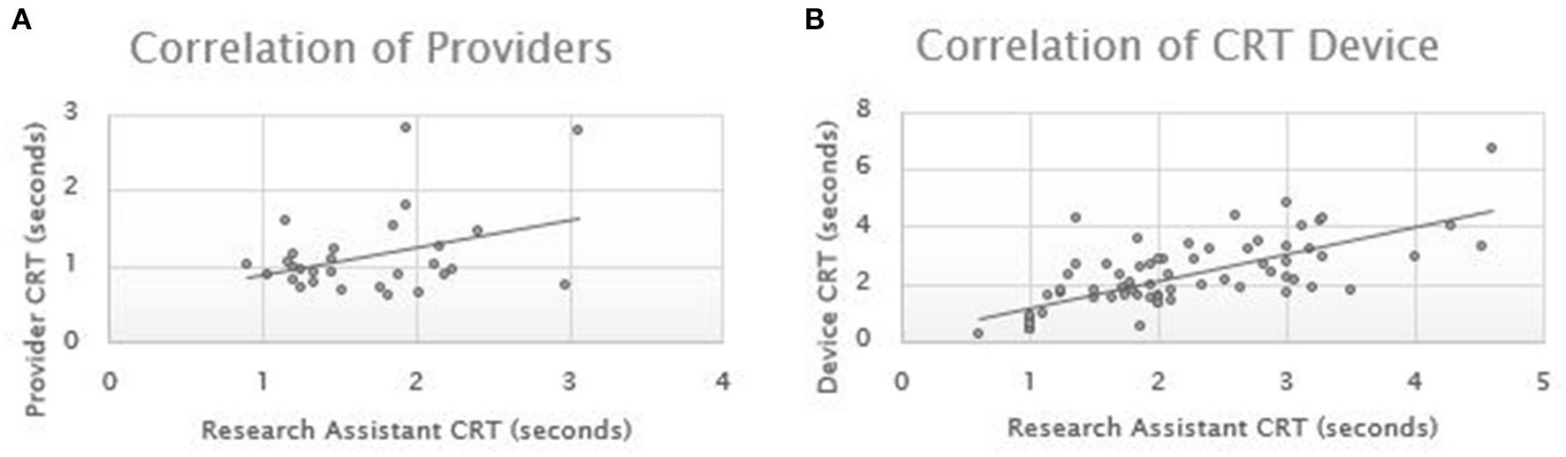
Capillary refill time correlation. CRT, capillary refill time.

Pearson correlations coefficients were performed for each group of measurements. The ED cohort consisted of 29 patients with an average age of 46.2 years (stdev 15.1) and 41% male. The ICU consisted of 25 patients with an average age of 58.5 years (stdev 16.1) and 40% male. The ICU patients had sequential CRT over their admission resulting in a total of 69 measurements. The Pearson correlation for the device CRT compared to research personnel CRT was 0.693. The Pearson correlation for the provider CRT compared to research personnel CRT was 0.359 (Figure 2).

## Discussion

There is growing body of research examining the role of bedside peripheral perfusion monitoring via CRT to direct diagnosis and therapeutic interventions for sepsis and shock. This represents a paradigm shift away from serum testing and invasive monitoring; while these will remain crucial, the non-invasive nature and immediacy of CRT make it appealing in the diagnosis and treatment of a dynamic disease process like sepsis. Prior reviews have detailed the promise of peripheral perfusion monitoring with technology in shock states to improve outcomes (5).

There is potential for this technology to be integrated into standard hospital monitoring protocols to identify decompensating patients earlier. One recent study performed across 3 institutions examined 6,500 hospital rapid response team activations (6). The authors noted CRT was an independent predictor of death, need for ICU transfer, and cardiac arrest. The ANDROMEDA shock trial randomized septic ICU patients to having their initial resuscitation guided by either CRT or blood lactate levels (7). The CRT group's outcomes were more favorable, in almost every category of morbidity and mortality, compared to the blood lactate group. Although it did not reach statistical significance (*p* = 0.06), when utilizing a pragmatic Bayesian analysis CRT was superior (8). A more recent study evaluated objective CRT measurement through a transmitted pulse oximetry light source and were able to show it significantly improved the test characteristics of standard sepsis screening scores in the ED (9). This type of technology has great promise for ED triage where studies have shown the ability to accurately triage sepsis patients can have significant impact on outcomes (10, 11).

This study had a number of limitations. The first is that there is no gold standard for CRT assessment to date. However, the best objective measure to date has been when CRT is performed by rigorous research personnel. For this reason this study used research assistants as the gold standard to compare the device to. Many factors can influence CRT including position, skin temperature, skin color, and hemoglobin concentration. However, the same protocol was used for each patient. In addition CRT were not compared to one another, but rather the CRT on the same patient using the device and gold standard. We believe this limits any confounding that would have been seen due to a patient differences, but future studies should evaluate these factors in further depth as we did not. A second limitation was the study was performed in two different hospital settings. However, this was done as capillary refill is measured in multiple places within healthcare and so the authors felt this was a pragmatic design. A single research assistant performed the manual and device measurements limiting the ability to assess inter-rater reliability. A third limitation is that this study was performed by a group that developed the technology. However, all study data was collected by research staff unrelated to the technology development. Further studies should be undertaken at multiple hospitals and will need to correlate CRT to outcomes including sepsis diagnosis, shock, lactate levels, and mortality.

This study demonstrates that CRT measured via light reflectance technology, is more highly correlated to a rigorous research standard measure than unstructured clinical measurement. This indicates the technology is a promising way to easily measure CRT in a rigorous and reproducible manner. Peripheral perfusion assessment by light reflectance has the potential to improve the care of sepsis and other shock states using point-of-care technology at the bedside.

## Data Availability Statement

The raw data supporting the conclusions of this article will be made available by the authors, without undue reservation.

## Ethics Statement

The studies involving human participants were reviewed and approved by Oregon Health & Science University. The patients/participants provided their written informed consent to participate in this study.

## Author Contributions

DS designed the study, data analysis, and drafted the manuscript. RC, RS, and MH revised it critically for important intellectual content. DS, RC, RS, and MH provide approval for publication of the content and agree to be accountable for all aspects of the work in ensuring that questions related to the accuracy or integrity of any part of the work are appropriately investigated and resolved. All authors contributed to the article and approved the submitted version.

## Conflict of Interest

The authors declare that this study received funding from Promedix Inc. The funder had the following involvement in the study: study design and data analysis.
